# SGLT2 Inhibitors in Kidney Diseases—A Narrative Review

**DOI:** 10.3390/ijms25094959

**Published:** 2024-05-01

**Authors:** Agata Gajewska, Jakub Wasiak, Natalia Sapeda, Ewelina Młynarska, Jacek Rysz, Beata Franczyk

**Affiliations:** 1 Department of Nephrocardiology, Medical University of Lodz, ul. Zeromskiego 113, 90-549 Lodz, Poland; agata.gajewska@stud.umed.lodz.pl (A.G.); jakub.wasiak@stud.umed.lodz.pl (J.W.); natalia.sapeda@stud.umed.lodz.pl (N.S.);; 2 Department of Nephrology, Hypertension and Family Medicine, Medical University of Lodz, ul. Zeromskiego 113, 90-549 Lodz, Poland

**Keywords:** SGLT2 inhibitors, chronic kidney disease, acute kidney injury, nephroprotection

## Abstract

Some of the most common conditions affecting people are kidney diseases. Among them, we distinguish chronic kidney disease and acute kidney injury. Both entities pose serious health risks, so new drugs are still being sought to treat and prevent them. In recent years, such a role has begun to be assigned to sodium-glucose cotransporter-2 (SGLT2) inhibitors. They increase the amount of glucose excreted in the urine. For this reason, they are currently used as a first-line drug in type 2 diabetes mellitus. Due to their demonstrated cardioprotective effect, they are also used in heart failure treatment. As for the renal effects of SGLT2 inhibitors, they reduce intraglomerular pressure and decrease albuminuria. This results in a slower decline in glomelular filtration rate (GFR) in patients with kidney disease. In addition, these drugs have anti-inflammatory and antifibrotic effects. In the following article, we review the evidence for the effectiveness of this group of drugs in kidney disease and their nephroprotective effect. Further research is still needed, but meta-analyses indicate SGLT2 inhibitors’ efficacy in kidney disease, especially the one caused by diabetes. Development of new drugs and clinical trials on specific patient subgroups will further refine their nephroprotective effects.

## 1. Introduction

Kidney disease represents a significant public health problem globally due to its prevalence, impact on individuals and communities, and the associated economic burden. Kidney disease, especially chronic kidney disease (CKD), is widespread worldwide [1]. 

CKD is a progressive disorder that affects more than 10% of the world’s population, which refers to over 800 million people [2], and this number has been increasing due to the growth in risk factors such as obesity and diabetes mellitus. An adult patient is diagnosed with CKD if their glomerular filtration rate (GFR) is less than 60 mL/min/1.73 m^2^ or greater than 60 mL/min/1.73 m^2^ and there is evidence of renal structural damage for three months or longer. Some markers of renal injury are albuminuria, structural changes detected in renal imaging, urine sediment abnormalities, electrolyte, and other abnormalities due to tubular disorders, histological changes in kidney biopsy, and previous kidney transplantation history. Chronic kidney disease can have various causes, and it often develops over an extended period. The most frequent causes of CKD are diabetes mellitus, hypertension, chronic glomerulonephritis, obstructive nephropathy, intestinal nephritis recurrent kidney infections, prolonged acute renal disease, and long-term use of certain medications. Individuals with early-stage CKD sometimes do not exhibit any apparent symptoms. This is why CKD is often referred to as a “silent” disease. As CKD progresses to more advanced stages, symptoms tend to become more pronounced and additional complications may arise. The most common symptoms of developed CKD include fatigue and weakness, edema, changes in urine frequency and volume, increased blood pressure, loss of appetite, and weight loss. CKD can be classified into five stages based on GFR, and three stages based on albuminuria. The treatment of CKD focuses on managing underlying causes, slowing the progression of the disease, and addressing complications to enhance quality of life. Treatment strategies may differ depending on the specific stage of CKD and the individual’s health status [3]. 

Acute kidney injury (AKI) is a sudden and often reversible decline in kidney function. AKI is defined by an abrupt increase in serum creatinine level (≥0.3 mg/dL within 48 h or ≥1.5 times baseline within 7 days) or a decrease in urine output <0.5 mL/kg/h for more than 6 h. AKI can be caused by a number of factors that impair the kidney’s normal functioning. These reasons are essentially divided into three categories: prerenal, intrinsic renal, and postrenal. While this classification may be helpful in differential diagnosis, AKI is a complex condition with shared pathophysiologic symptoms across all categories. The breakdown of the causes of AKI is shown in Table 1 (Turgut et al., 2023 [4]).

Acute kidney injury symptoms can range in severity and appear differently in various people. The symptoms of AKI frequently arise suddenly and may include oliguria or anuria, edema, fatigue and weakness, shortness of breath, disorientation and altered mental status, nausea and vomiting, hypertension or hypotension, and elevated heart rate. AKI is commonly classified into three stages based on the Kidney Disease: Improving Global Outcomes (KDIGO) criteria, which consider changes in serum creatinine levels and urine output. The treatment options for AKI are limited and primarily supportive. The clinical strategy should begin with assessing hemodynamic stability, identifying signs of AKI consequences, determining the cause, and treating the condition [5]. Short-term consequences of AKI include longer hospital stays, higher medical expenses, and mortality during hospital stays. AKI can also lead to long-term complications, including cardiovascular events, progression to CKD, and increased mortality [6]. 

Recent studies suggest that these two syndromes are not distinct entities but rather are closely interconnected. CKD acts as a risk factor for the occurrence of AKI, while AKI serves as a risk factor for the onset of CKD. Additionally, both AKI and CKD pose risks for the development of cardiovascular disease [7]. This emphasizes the necessity of investigating therapeutic targets in the context of renal diseases, which may contribute to the development of new and effective medications. In recent years, there has been a lot of interest in studying sodium-glucose cotransporter-2 (SGLT2) inhibitors in the setting of kidney diseases [8]. This review discusses emerging evidence supporting the therapeutic use of SGLT2 inhibitors in patients with both CKD and AKI.

## 2. SGLT2 Inhibitors, Their Mechanism of Action, and Effects on Kidneys

Sodium-glucose cotransporter-2 inhibitors are a new group of drugs that became available after 2010, with their continuous rise of popularity reaching its peak today. The precursor to this group of drugs was phlorizin, an o-aryl glucoside of plant origin, discovered in the 19th century and found to be a nonselective sodium-glucose cotransporter inhibitor. Unfortunately, due to poor oral bioavailability and gastrointestinal adverse effects, this substance never reached the human trial stage [9]. As has been shown, c-glucosides, discovered in the second half of the 20th century, have better bioavailability and significantly fewer adverse effects [10]. The first drug of this group approved for use in the European Union was dapagliflozin in 2012. In the United States, on the other hand, the first SGLT2 inhibitor approved for use was canagliflozin in 2013 [11,12]. Due to their mechanism of action, these substances were initially considered primarily antidiabetic drugs; however, with deepening research, additional properties of these drugs have been demonstrated. In 2020, the United States Food and Drug Administration (US FDA) expanded the indication for dapagliflozin to include the treatment of heart failure with reduced ejection fraction and then, also, for patients with preserved ejection fraction [13]. In addition, the use of dapagliflozin in CKD to slow the progression of this disease was approved in 2021 [14]. There are now more drugs from this group approved for use, and the most popular include drugs such as empagliflozin, dapagliflozin, canagliflozin, or ertugliflozin [15]. 

**Figure 1:**
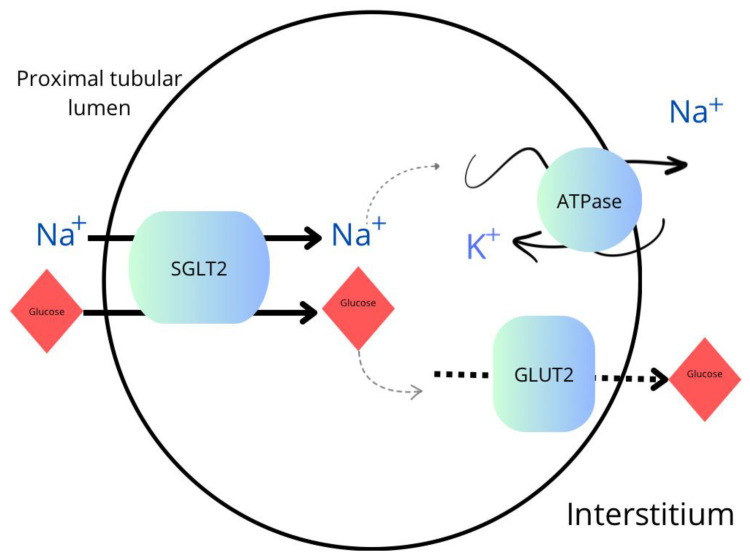
Transport of sodium and glucose in the proximal tubule. SGLT2—sodium-glucose cotransporter-2 inhibitor; GLUT2—glucose transporter 2; ATPase—sodium–potassium adenosine triphosphatase.

SGLT2 inhibitors act as specific antagonists of SGLT2 transporters, which are integral membrane proteins mainly found in the proximal tubules of the kidneys. These inhibitors bind to the SGLT2 protein, inhibiting its function and preventing the reabsorption of glucose from urine back into the bloodstream. The SGLT2 protein is responsible for the covalent binding of sodium and glucose in the proximal tubules of the kidneys. This process occurs in a 1:1 ratio, meaning that each sodium ion is transported together with one molecule of glucose. It is a simultaneous process. The main site of action of SGLT2 is the luminal part of the proximal tubules where this transporter is present on the cell membrane, enabling the active transport of sodium and glucose from urine into the cell interior. Those inhibitors bind to the active site of SGLT2, which includes the binding site for glucose and sodium. Through this binding, inhibitors form complexes with the SGLT2 protein, preventing the active transport of glucose and sodium. As a result, glucose remains in the renal tubule and is excreted from the body, leading to a decrease in blood glucose concentration. This mechanism is shown schematically in Figure 1 [16,17,18].

Another sodium-dependent glucose transporter is SGLT1 found mainly in the intestine. However, the drugs in question are 200–250 times more selective for SGLT2 than for SGLT1 [15]. Normally SGLT2 is responsible for the reabsorption of about 90% of the glucose filtered by the kidneys, and in normoglycemic individuals, it is about 140–160 g of glucose each day [19]. This way, the kidneys contribute to maintaining glucose homeostasis in the body. However, when blood glucose levels are elevated in both type 1 and type 2 diabetes, there is a significant increase in reabsorption capacity and the amount of glucose reabsorbed into the bloodstream. This increase is possible up to a blood glucose level of as much as 12 mmol/L, after which the reabsorption capacity is disturbed and glucosuria occurs. These mechanisms result in persistent hyperglycemia in diabetic patients [20]. SGLT2 inhibitors exert a range of beneficial clinical effects on the kidneys. One of the most important is a decrease in glomerular hyperfiltration. A key process in the kidney that controls the GFR to preserve renal function and fluid–electrolyte balance is known as tubuloglomerular feedback (TGF). This feedback system interacts precisely between the tubular and glomerular segments of the nephron, allowing the kidney to dynamically modify filtration in response to intrarenal changes. The TGF mechanism starts at the glomerulus where blood is filtered to generate a filtrate that then reaches the nephron’s proximal tubule. As the filtrate passes through the nephron, it ultimately reaches the macula densa, a specialized part of the distal convoluted tubule. This group of cells is strategically located at the point where the distal tubule meets the afferent arteriole, which supplies blood to the glomerulus. The macula densa cells are essential for TGF because they can detect sodium chloride (NaCl) levels present in the filtrate, which presents essential information about the present condition of glomerular filtration. Elevated NaCl concentrations indicate either an overly high filtration rate or a reduction in the nephron’s reabsorption efficiency. The macula densa cells react to this signal by causing vasoconstriction in the afferent arteriole. This constriction decreases blood flow to the glomerulus, lowering the GFR and restoring equilibrium. Conversely, when NaCl concentrations in the filtrate are low, indicating a slower filtration rate or higher reabsorption, the macula densa cells provide a signal to dilate the afferent arteriole. This dilatation increases blood flow to the glomerulus, raising the GFR and compensating for decreased filtration. Through these feedback systems, the kidney may better maintain homeostasis and provide the best conditions for reabsorption and waste excretion [21,22]. 

Glomerular hyperfiltration is a condition in which there is an unusually high rate of blood flow through the glomeruli, resulting in excessive production of pro-urine [23]. It is frequently seen as an early sign of diabetic kidney disease (DKD) and is linked to the development of kidney damage [24]. These medications increase the amount of glucose excreted in urine by blocking the ability of SGLT2 protein in the proximal tubules to reabsorb glucose. This process results in natriuresis and osmotic diuresis, which, in turn, reduces glomerular hyperfiltration, which can slow down the development of DKD [25].

Reduction in albuminuria is another significant clinical effect of SGLT2 inhibitors on the kidneys [26]. Albuminuria, defined as the presence of excess albumin in the urine, is a sign of kidney impairment and is frequently linked to the development of cardiovascular events and more severe renal dysfunction [27]. Increased intraglomerular pressure is linked to impairment of the glomerular filtration barrier, resulting in increased permeability to albumin and other proteins [28]. SGLT2 inhibitors contribute to the normalization of glomerular pressure by lowering glomerular hyperfiltration, as previously discussed. Normalizing this pressure helps to reduce albumin leakage into urine [29]. Another condition linked to increased vascular permeability and albumin leakage into the urine is endothelial dysfunction [30]. It has been demonstrated that SGLT2 inhibitors improve endothelial function, which can result in reduced albuminuria [31]. 

In addition to their glucose-lowering properties, SGLT2 inhibitors have been shown to have direct renoprotective effects. It has been proposed that the most important mechanisms include the reduction in intraglomerular pressure and the reduction of albuminuria in patients with diabetes-associated kidney disease. Researchers suggest additional mechanisms that may be involved in that process, such as oxidative stress reduction and anti-inflammatory and antifibrotic effects.

An important factor in the development of kidney damage in many renal illnesses, including AKI and CKD, is chronic inflammation [32]. It has been demonstrated that SGLT2 inhibitors have anti-inflammatory qualities, assisting in lowering renal inflammation. These drugs can inhibit the production of pro-inflammatory chemokines and cytokines like interleukin-1 (IL-1) [33] and interleukin-6 (IL-6) [34]. SGLT2 inhibitors help to reduce the inflammatory response in the kidneys by decreasing various inflammatory signaling pathways, which preserve renal structure and function [35]. First, they induce the activation of AMPK, an essential regulator of the metabolism of cellular energy, which then suppresses the NF-κB and MAPK pathways, thereby reducing the synthesis of inflammatory mediators. Second, SGLT2 inhibitors upregulate SIRT1, a NAD+-dependent deacetylase known for its anti-inflammatory effects, which further attenuates inflammation by inhibiting NF-κB activity. Furthermore, these inhibitors induce the expression of PGC-1α, a key regulator of oxidative metabolism and mitochondrial biogenesis, which improves mitochondrial function, reduces oxidative stress, and mitigates inflammation [36].

A degenerative process known as renal fibrosis is defined by an excessive accumulation of extracellular matrix proteins, mostly collagen, in the renal interstitium. The process is frequently seen in the later stages of renal disease [37]. It has been demonstrated that SGLT2 inhibitors have antifibrotic effects in the kidneys, which contributes to their renoprotective qualities [38]. The therapeutic effect of SGLT2 inhibitors on renal fibrosis may be achieved through multiple pathways regulation, but they are not fully understood [39]. In trials, SGLT2 inhibitors have been shown to reduce inflammatory markers such as IL-6, TNF, IFN-γ, NF-κβ, TLR-4, and TGF-β, increase mitochondrial function, and limit mesangial and myofibroblast growth [34,40]. Furthermore, Guo et al. found that in streptozotocin-induced diabetic nephropathy models, mice treated with dapagliflozin exhibited remission of conditions such as glomerular sclerosis, thickening of the glomerular basement membrane (GBM), and podocyte damage in the glomeruli—all of which are associated with renal sclerosis. The proposed mechanism involves SGLT2 inhibitors suppressing epithelial-mesenchymal transition in podocytes through downregulation of the IGF1R/PI3K pathway. In diabetes, overexpression of SGLT2 in podocytes creates an intracellular hyperglycemic environment, enhancing IGF1R/PI3K signaling. At the molecular level, increased levels of IGF1 and IGF2 bind closely to IGF1 receptors, leading to enhanced phosphorylation of PI3K and subsequent podocyte dysfunction, including enhanced epithelial-mesenchymal transition (EMT). This molecular imbalance results in podocyte loss and renal fibrosis. Inhibition of the SGLT2 receptor by SGLT2 inhibitors decreased IGF1R/PI3K activity in both in vitro and in vivo experiments. This inhibition led to the attenuation of podocyte damage mediated by the IGF1R/PI3K signaling pathway, resulting in decreased circulating levels of IGF1 and IGF2. These findings suggest that podocytes are a significant target of SGLT2 inhibitors, providing a potential mechanism for their renoprotective and anti-proteinuria effects [41]. 

Renal fibrosis and inflammation are exacerbated by oxidative stress, which is triggered by an imbalance between antioxidant defenses and the creation of reactive oxygen species (ROS) [42]. SGLT2 inhibitors have been found to enhance the antioxidant capacity within the kidneys. These drugs promote the activity of antioxidant enzymes such as superoxide dismutase (SOD), catalase, and glutathione peroxidase, which help neutralize ROS and minimize oxidative damage to renal tissues [43]. SGLT2 inhibitors possess direct antioxidant capabilities and can operate as free radical scavengers, neutralizing ROS and preventing them from causing oxidative damage to renal cells [44]. Trials have demonstrated that dapagliflozin may ameliorate endothelial dysfunction through a variety of molecular mechanisms. It has been shown to restore the activity of endothelial nitric oxide synthase (eNOS), increasing the generation of nitric oxide (NO), a major regulator of vascular function. Furthermore, dapagliflozin has been demonstrated to improve NO bioavailability by blocking its degradation and supporting its release from endothelial cells, hence improving vasodilation and vascular function. These effects are primarily mediated by the activation of sirtuin 1 (SIRT1), which improves eNOS activity, NO production, and ROS inhibition, thereby enhancing endothelial function [36,45].

SGLT2 inhibitors have a variety of clinical effects on the kidneys and show promise for managing CKD and AKI. Potential advantages of SGLT2 inhibitors include preventing AKI and delaying the development of CKD.

## 3. Pleiotropic Effects of SGLT2 Inhibitors

When SGLT2 inhibitors are used, there is a definite rise in glucose excretion, dependent on the level of glucose in the blood. In normoglycemic individuals, there can be an increase of about 40–80 g/day. However, in diabetics, this number is higher [46,47,48]. As meta-analyses have shown, the use of these drugs results in a decrease in HbA1c levels by an average of 0.61% when it is added to other antidiabetic medication and by 0.79% in monotherapy [49]. In addition, the loss of glucose from urine results in the loss of calories. This has been proven to result in weight loss in patients [50]. Moreover, a statistically significant decrease in systolic blood pressure by about 3–4 mmHg and diastolic blood pressure by 1–2 mmHg was also observed [51]. A definite advantage of SGLT2 inhibitors is that they are independent of insulin secretion and do not increase the risk of hypoglycemia in monotherapy [52]. Currently, these drugs are allowed as first-line treatment for monotherapy in type 2 diabetes [53]. Worth noting, however, are the most common adverse effects of these drugs. It has been shown that SGLT2 inhibitors significantly increase the risk of diabetic ketoacidosis and volume depletion and, therefore, must be used with caution with other drugs affecting renal hemodynamics, especially in patients with extracellular volume depletion. The most common complication is urogenital infections, but these are usually mild in nature, and treatment with standard medications is sufficient. The use of these drugs has also been linked to an increased risk of bone fractures and limb amputations [6,15,54]. 

However, in addition to glucose, SGLT2 is also involved in the transport of metabolites such as ketones, especially β-hydroxybutyrate (β-HBA), and uric acid. Regarding the transport of ketones, this mechanism is not yet fully understood, but there are studies suggesting that SGLT2 inhibitors may influence the secretion of ketones from liver cells and increase their excretion in urine. For β-HBA, it has been observed that SGLT2 inhibitors may decrease its levels in the blood by increasing urinary excretion. As for uric acid, the SGLT2 inhibitors affect its transport through the renal tubules. Increased excretion of uric acid is beneficial for patients with gout or hyperuricemia. As a result, the action of SGLT2 inhibitors on metabolites other than glucose and sodium may be due to their interaction with the SGLT2 protein and their impact on transport processes in the kidneys. These mechanisms may have significant clinical implications, especially in the context of ketosis development and influence on purine metabolism [55].

Additionally, dapagliflozin may increase fatty acid oxidation in muscles, thereby reducing triglyceride levels. Empagliflozin inhibits cholesterol synthesis in the liver by reducing the activity of the enzyme HMG-CoA reductase (3-hydroxy-3-methylglutaryl-coenzyme A reductase), leading to a decrease in total cholesterol levels. Besides, dapagliflozin increases the level of HDL-C cholesterol by regulating the reverse cholesterol transport pathway through the expression of the protein. While the impact on LDL-C cholesterol levels is somewhat less clear, there is evidence of the reduction in its concentration in some patients using SGLT2 inhibitors, possibly due to the regulation of LDL receptor expression and increased removal of LDL from the blood. These mechanisms of lipid level influence may have significant implications for the metabolic health of patients [56].

With the development of research on SGLT2, its positive effects on the cardiovascular (CV) system soon began to be noticed. As shown, empagliflozin significantly reduced the primary endpoint defined as one of CV death, nonfatal myocardial infarction, and nonfatal stroke in type 2 diabetic patients. There was a 38% decrease in CV deaths when adding this drug to diabetes therapy [57]. In addition to the previously mentioned drop in blood pressure or body weight, this is probably due to osmotic diuresis, leading to a reduction in cardiac preload and, thus, reducing the symptoms of heart failure [49]. This is confirmed by the 9% decrease in plasma volume with the use of dapagliflozin [58]. 

**Figure 2:**
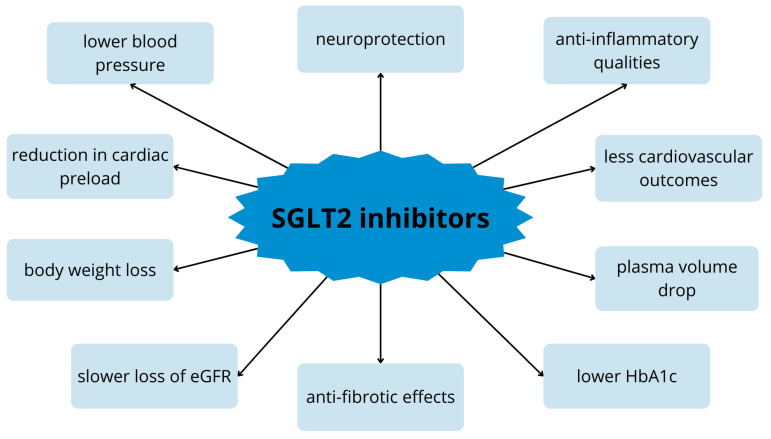
Pleiotropic effect of SGLT2 inhibitors on the body. eGFR—estimated glomelural filtration; HbA1c—glycated hemoglobin.

Importantly, multicenter studies and meta-analyses confirmed that SGLT2 inhibitors also reduce the risk of CV outcome in patients without diabetes [59,60,61]. Given these reports, clinical practice guidelines now recommend the use of SGLT2 inhibitors in patients with heart failure across the spectrum of ejection fraction and in combination with various other drugs [13,62]. As previously described, flozins have anti-inflammatory and antioxidant properties and improve epithelial function in both the kidney and nervous system. SGLT2 inhibitors can also inhibit acetylcholinesterase, which contributes to cognitive improvement. These properties speak of their neuroprotective effect [63]. The pleiotropic properties of these drugs are shown in Figure 2 [64].

## 4. Evidence of the Efficiency of SGLT2 Inhibitors in Kidney Diseases

CKD is an independent risk factor for cardiovascular disease. Furthermore, a decline in GFR is a direct cause of an increased cardiovascular mortality risk. Therefore, it is justified to seek methods that improve renal function and performance [65]. We observe a series of conducted studies that demonstrate cardiovascular and renal benefits associated with the use of SGLT2 inhibitors [66].

CKD is recognized to be associated with chronic tubular hypoxia. SGLT2 inhibitors, by reducing the burden on the proximal tubules, ameliorate their hypoxia. One of the predisposing conditions to renal tubular hypoxia is diabetes. Hypoxia ensues from increased energy consumption in the proximal tubules due to enhanced glucose absorption [14,67].

Evidence of the nephroprotective effect of SGLT2 inhibitors is provided by a randomized trial involving 17,160 patients with type 2 diabetes. Participants in the study received either dapagliflozin or a placebo. The mean eGFR in the studied patients was 85.2 mL/min/1.73 m^2^, with 45% of patients presenting with eGFR between 60 mL/min/1.73 m^2^ and 90 mL/min/1.73 m^2^. At the initial stage of the study, 7% of patients had eGFR below 60 mL/min/1.73 m^2^. Renal events were observed in 4.3% of patients receiving actual dapagliflozin and 5.6% in patients receiving a placebo [68].

Subsequent research examining the effects of SGLT2 inhibitors involved a randomized trial encompassing 10,142 individuals with type 2 diabetes and a high cardiovascular risk. This time, patients were administered canagliflozin or a placebo. Renal outcomes were deemed statistically insignificant; however, a potential impact on albuminuria was documented. Reduction in albuminuria occurred more frequently in patients receiving canagliflozin compared with those receiving a placebo. Conversely, an increase in albuminuria occurred significantly less frequently in individuals receiving canagliflozin than in those on a placebo [69].

In one study, molecular models were analyzed to examine the impact of canagliflozin on biomarkers involved in DKD. Proteins were selected to facilitate the assessment of canagliflozin’s effects on DKD. Reduction in plasma levels of TNF receptor 1, matrix metalloproteinase 7, and fibronectin 1 suggests that canagliflozin may reduce fibrosis and inflammation in the kidneys [70].

Another study examining the impact of the SGLT2 inhibitor, specifically dapagliflozin, is a long-term study of patients with type 2 diabetes and moderate renal impairment. In patients, in addition to changes in glycated hemoglobin levels and weight loss, an initial decrease in mean eGFR and creatinine clearance was observed after one week of dapagliflozin treatment. Possibly, it was due to the slight antihypertensive or diuretic effect. However, subsequently, stabilization of mean eGFR and creatinine clearance was noted, unlike in the placebo group where these values decreased [71].

Likewise, in a randomized trial involving patients with type 2 diabetes and estimated eGFR of at least 30 mL/min/1.73 m^2^ who received empagliflozin or a placebo, it was found that empagliflozin administration was associated with slower progression of kidney disease. The exacerbation of nephropathy transpired in 12.7% of patients receiving empagliflozin, whereas in those receiving a placebo, it was 18.8%. Furthermore, in patients receiving empagliflozin, the doubling of creatinine levels occurred in 70 out of 4645 patients, yielding a rate of 1.5%. In contrast, in the placebo group, a doubling of creatinine levels took place in 60 out of 2323 individuals, constituting 2.6%. The addition of empagliflozin to the standard therapy of these patients reduced the frequency of renal events, improved kidney function, and delayed the need for renal replacement therapy [72].

In a double-blind trial, 3730 patients with heart failure (HF) were allocated to receive either empagliflozin (10 mg once daily) or a placebo. The study meticulously examined the impact of an SGLT2 inhibitor on the cardiovascular system. However, this is another instance where a reduction in the rate of decline of the estimated glomerular filtration rate was observed in patients receiving empagliflozin compared with those receiving a placebo. Additionally, through this study, the nephroprotective effect of the investigated drugs can be discerned [73].

Currently, a superior effect of dapagliflozin compared with empagliflozin is noted in many studies. This is, of course, associated with the conduct of numerous large-scale meta-analyses, including a meta-analysis comparing the effects of these two inhibitors in patients with type 2 diabetes at risk of atrial fibrillation occurrence [74]. It should be noted that there is a causal relationship between CKD and other diseases, including HF [75]. In patients with CKD and diabetes, the risk of cardiovascular complications increases more rapidly than in patients without diabetes [76]. Therefore, it is difficult to analyze the direct impact of SGLT2 inhibitors only on chronic kidney disease. The recently published DAPA-CKD study (Dapagliflozin and Prevention of Adverse Outcomes in Chronic Kidney Disease) focused on diabetic or nondiabetic CKD treated with dapagliflozin. This randomized study involved 4304 participants receiving dapagliflozin or a placebo. The study demonstrated a reduction in the likelihood of hospitalization due to HF in patients without previous symptoms of HF. Moreover, the decline in eGFR was significantly slower in patients receiving dapagliflozin compared with those receiving a placebo in both diabetic and nondiabetic patients [77]. The DAPA-CKD study is one of the frequently cited trials, and the results obtained during this research have been incorporated into many guidelines, indicating its reliability. The summary of the described studies is presented in Table 2. Regarding acute kidney injury, clinical studies paradoxically did not demonstrate a heightened risk of AKI during the administration of SGLT2 inhibitor therapy. Nevertheless, relevant caution is advised regarding the continuation of therapy when patients exhibit a diminished eGFR below 45 mL/min/1.73 m^2^. This is justified due to the previously mentioned mechanism of action of these drugs, which initially leads to a reduction in eGFR [78]. Anticipating an enhanced risk of AKI in patients taking SGLT2 inhibitors, a large-scale meta-analysis was conducted to elucidate this matter. The frequency of AKI incidents in patients on SGLT2 inhibitors was compared to those receiving a placebo. A decline in the rate of AKI incidents was noted among patients receiving the discussed medications [79]. One meta-analysis even suggested that SGLT2 inhibitors significantly reduce the risk of AKI occurrence [80].

## 5. Conclusions, Future Perspectives, and Limitations

Research and clinical interest in the potential uses of SGLT2 inhibitors in renal disease is constantly evolving. The future of SGLT2 inhibitors in the context of kidney health holds several promising directions and considerations. 

First, one line of inquiry is the creation of novel SGLT2 inhibitors, emphasizing improving efficacy, optimizing safety profiles, and developing novel mechanisms of action. To maximize therapeutic results, this may include changes in chemical structures and innovative approaches to drug delivery. Ongoing research in these areas attempts to improve our understanding of the complex mechanisms by which SGLT2 inhibitors work, providing vital insights into their potential therapeutic applications. Furthermore, scientists might investigate combination treatments, which involve administering SGLT2 inhibitors along with other drug classes. That would enable us to address several pathways related to kidney disease, promoting a more comprehensive and integrative therapeutic approach. 

Additional investigation into defining the precise patient subgroup that would benefit most from these drugs is another direction for the future. Customizing therapies according to patient characteristics would increase the potential for successful outcomes. Researchers should also continue investigating early intervention with SGLT2 inhibitors to get insights into the best timing, patient selection, and long-term outcomes. Furthermore, SGLT2 inhibitor use may expand beyond the regular adult population, with studies exploring their safety and efficacy in adolescent kidney disease patients. Moreover, specific populations, such as pregnant women or individuals with specific comorbidities, may become the objects of targeted research. 

The financial implications of SGLT2 inhibitors will probably become more of a matter of attention and discussion in the medical community as new evidence and guidelines are developed. The economic benefits of SGLT2 inhibitors are particularly notable due to their positive impact on cardiovascular health. By reducing heart failure hospitalizations [81], these medications can contribute to cost savings related to hospital stays and subsequent medical expenses. Furthermore, the demonstrated renal protective benefits of SGLT2 inhibitors, which decrease the progression of kidney disease [8], have possible economic implications. This relates to the potential reduction in costly therapies associated with advanced renal disease stages, such as dialysis or kidney transplantation. The role of SGLT2 inhibitors in influencing healthcare resource utilization is a pivotal aspect of their economic profile. While these medications have some initial expenses, their long-term advantages have the potential to result in significant cost reductions. The positive effects of SGLT2 inhibitors on patients’ quality of life may result in higher productivity and lower indirect expenses related to impairment. That is why global accessibility and affordability of SGLT2 inhibitors are crucial economic factors. 

The evidence from practical applications will persist in enhancing the conclusions derived from clinical studies, offering significant perspectives on the pragmatic application and results of SGLT2 inhibitors in many contexts. Maintaining long-term safety monitoring guarantees continuous evaluation of potential risks or adverse effects related to prolonged usage. It is also imperative that clinical recommendations are updated and incorporated by the latest research on the use of SGLT2 inhibitors in kidney disease as the area develops. 

Although SGLT2 inhibitors have become essential medications for the treatment of diabetic kidney disease, administering them in certain patients requires careful assessment of any possible limitations. 

Firstly, the pharmacokinetics of SGLT2 inhibitors are directly related to renal function. These drugs are mostly eliminated by the kidneys, with limited hepatic metabolism. As a result, insufficient renal function, as observed in CKD or end-stage renal disease, reduces medication clearance and increases the possibility of drug accumulation. In individuals with renal impairment, careful dose modifications are necessary to reduce the risk of drug accumulation and related side effects. The use of SGLT2 inhibitors may be contraindicated in situations of severe kidney dysfunction, especially in patients requiring dialysis for end-stage renal disease, due to the difficulties in achieving proper dosage and maintaining therapeutic plasma concentrations [82].

The reduced glomerular filtration rate (GFR) associated with renal impairment could negatively impact the effectiveness of SGLT2 inhibitors in lowering blood glucose levels. As a result, individuals with severe kidney dysfunction may not benefit from SGLT2 inhibitors to the same extent as those with normal renal function in terms of glycemic control. This emphasizes the significance of integrating extra therapeutic approaches in the treatment of patients with renal impairment, such as insulin therapy or other drugs that decrease blood glucose levels.

The safety profile of SGLT2 inhibitors in individuals with renal impairment requires careful evaluation due to several factors. SGLT2 inhibitors induce electrolyte abnormalities, particularly pronounced in patients with kidney disease. These drugs enhance glycosuria by blocking the sodium-glucose cotransporter-2 (SGLT2) in the proximal tubule of the nephron, which increases urine glucose excretion along with sodium and water. Several studies have indicated mild increases in serum potassium concentrations with SGLT2 inhibitor medication. Despite a minor rise in serum potassium concentration, individuals with renal impairment or those on medications that enhance the risk of hyperkalemia, such as RAAS inhibitors, should have their levels closely monitored [83]. In a meta-analysis conducted by Tang and Zhang, which encompassed 18 randomized controlled trials involving 15,309 patients treated with four different SGLT2 inhibitors (canagliflozin, empagliflozin, dapagliflozin, and ipragliflozin), it was demonstrated that these drugs can elevate serum magnesium levels by approximately 0.08 to 0.2 mEq/L in diabetic patients lacking normal renal function. Notably, the study revealed an intriguing finding regarding canagliflozin, which exhibits a linear and dose-dependent effect in increasing serum magnesium levels [84]. Additionally, the meta-analysis by Rong et al. revealed an increased risk of hypovolemia with SGLT2 inhibitors in patients with type 2 diabetes mellitus (T2DM). This emphasizes the need to be cautious, especially with older individuals and those with moderate renal impairment as they may be more susceptible to this adverse effect [85].

Although findings are still not conclusive, it is crucial to note that a growing body of evidence suggests that using SGLT2 inhibitors may have some effects on bone health and the mineral metabolism index. In a study conducted by Balu et al., treatment with canagliflozin resulted in notable changes in several key markers of mineral metabolism compared with controls. Specifically, compared with a placebo, canagliflozin increased serum phosphorus levels by 16%, plasma fibroblast growth factor 23 (FGF23) by 20%, and plasma parathyroid hormone (PTH) by 25%. Conversely, the administration of canagliflozin led to a decrease of 10% in plasma 1.25-dihydroxyvitamin D levels. These alterations point to an effect of SGLT2 inhibitors on mineral metabolism, but more research is required to completely comprehend these pathways [86].

SGLT2 inhibitors are also associated with increased risks of genital mycotic infections, particularly in individuals with a prior history of such infections. Additionally, they elevate the risk of uncomplicated urinary tract infections (UTI), urosepsis, or pyelonephritis. While the exact mechanism behind UTIs linked to SGLT2 inhibitors remains unclear, the glucosuria induced by these medications may create a conducive environment for microbial growth, potentially exacerbating infection risk [87].

In conclusion, the future implications for SGLT2 inhibitors in renal health represent a comprehensive approach, providing new opportunities for prevention, intervention, and improved patient outcomes in the field of kidney-related diseases. Overall, while these medications provide significant benefits, clinicians should consider individual patient factors and potential limitations when prescribing SGLT2 inhibitors in kidney disease. Regular monitoring and patient education are crucial components of safe and effective SGLT2 inhibitor therapy in kidney disease.

## Figures and Tables

**Table 1:** Summary of exemplary causes of prerenal, renal, and postrenal AKI.

Categories of AKI	Causes
Prerenal AKI	low blood volume, low blood pressure, heart failure, hepatorenal syndrome, and local alterations to the blood vessels supplying the kidney
Renal AKI	glomerulonephritis, acute tubular necrosis (ATN), infections, acute interstitial nephritis, rhabdomyolysis, tumor lysis syndrome, inflammatory conditions, or the use of certain medications
Postrenal AKI	benign prostatic hyperplasia, kidney stones, urethral obstruction, bladder stones, or tumors that restrict urine flow

**Table 2:** Comparison of clinical studies on SGLT2 inhibitors in renal protection and diabetes management.

Study	Object of the Study	Main Findings	Positive Outcomes	Negative Outcomes	Uncertain Outcomes
Wiviott et al. [68]	Cardiovascular effects of Dapagliflozin in type 2 diabetes patients	Dapagliflozin reduces the risk of cardiovascular events	Reduction in cardiovascular risk	No improvement in glycemic control	Short follow-up period, potential for unmeasured confounders, dropout rate
Neal et al. [69]	Cardiovascular and renal effects of Canagliflozin in type 2 diabetes patients	Canagliflozin reduces the risk of cardiovascular and renal events	Reduction in cardiovascular and renal risk	-	Potential for unmeasured confounders
Heerspink et al. [67]	Renoprotective effects of sodium-glucose cotransporter-2 inhibitors	SGLT2 inhibitors show a beneficial effect on the kidneys	Kidney protection	-	Short follow-up period, potential for unmeasured confounders, small sample size
Kohan et al. [71]	Long-term study of patients with type 2 diabetes and moderate renal impairment	Dapagliflozin reduces weight and blood pressure but does not improve glycemic control	Weight and blood pressure reduction	No improvement in glycemic control	Short follow-up period, potential for unmeasured confounders, small sample size
McMurray et al. [77]	Effects of Dapagliflozin in patients with kidney disease, with and without heart failure	Dapagliflozin has beneficial effects on patients with kidney disease, with and without heart failure	Benefits for patients with kidney disease and/or heart failure	-	Short follow-up period, potential for unmeasured confounders, dropout rate

SGLT2 inhibitors—sodium-glucose cotransporter-2 inhibitors.

## Data Availability

The data presented in this study are available upon request from the correspondent author due to the partial obtaining of articles within time limits. Full versions for access from the corresponding author.
